# Autophagy activity is associated with membranous sodium iodide symporter expression and clinical response to radioiodine therapy in non-medullary thyroid cancer

**DOI:** 10.1080/15548627.2016.1174802

**Published:** 2016-04-22

**Authors:** Theo S. Plantinga, Marika H. Tesselaar, Hans Morreau, Eleonora P. M. Corssmit, Brigith K. Willemsen, Benno Kusters, A. C. H. van Engen-van Grunsven, Johannes W. A. Smit, Romana T. Netea-Maier

**Affiliations:** a Department of Internal Medicine, Radboud University Medical Center, Nijmegen, The Netherlands; b Division of Endocrinology, Radboud University Medical Center, Nijmegen, The Netherlands; c Department of Pathology, Radboud University Medical Center, Nijmegen, The Netherlands; d Department of Pathology, Leiden University Medical Center, Leiden, The Netherlands; e Department of Endocrinology and Metabolic Diseases, Leiden University Medical Center, Leiden, The Netherlands

**Keywords:** autophagy, LC3, puncta, radioactive iodine therapy, thyroid carcinoma

## Abstract

Although non-medullary thyroid cancer (NMTC) generally has a good prognosis, 30–40% of patients with distant metastases develop resistance to radioactive iodine (RAI) therapy due to tumor dedifferentiation. For these patients, treatment options are limited and prognosis is poor. In the present study, expression and activity of autophagy was assessed in large sets of normal, benign and malignant tissues and was correlated with pathology, SLC5A5/hNIS (solute carrier family 5 member 5) protein expression, and with clinical response to RAI ablation therapy in NMTC patients. Fluorescent immunostaining for the autophagy marker LC3 was performed on 100 benign and 80 malignant thyroid tissues. Semiquantitative scoring was generated for both diffuse LC3-I intensity and number of LC3-II-positive puncta and was correlated with SLC5A5 protein expression and clinical parameters. Degree of diffuse LC3-I intensity and number of LC3-II-positive puncta scoring were not discriminative for benign vs. malignant thyroid lesions. Interestingly, however, in NMTC patients significant associations were observed between diffuse LC3-I intensity and LC3-II-positive puncta scoring on the one hand and clinical response to RAI therapy on the other hand (odds ratio [OR] = 3.13, 95% confidence interval [CI] =1.91–5.12, P = 0.01; OR = 5.68, 95%CI = 3.02–10.05, P = 0.002, respectively). Mechanistically, the number of LC3-II-positive puncta correlated with membranous SLC5A5 expression (OR = 7.71, 95%CI = 4.15–11.75, P<0.001), number of RAI treatments required to reach remission (P = 0.014), cumulative RAI dose (P = 0.026) and with overall remission and recurrence rates (P = 0.031). In conclusion, autophagy activity strongly correlates with clinical response of NMTC patients to RAI therapy, potentially by its capacity to maintain tumor cell differentiation and to preserve functional iodide uptake.

## Introduction

Patients diagnosed with non-medullary thyroid cancer (NMTC) are, in addition to determination of the histological subtype, regularly classified according to the differentiation status of the tumor, ranging from well-differentiated to severely dedifferentiated NMTC.^1,2^ The first-line therapeutic regimen consists of thyroidectomy followed by ablation of thyroid (tumor) remnants by ^131^I radioactive iodine therapy (RAI). Importantly, in about 30–40% of patients with metastatic disease this treatment strategy is not curative because of tumor cell resistance to RAI caused by a process of dedifferentiation and concomitant loss of thyroid-specific gene expression including the gene encoding the human sodium iodide symporter (SLC5A5), leaving patients at high risk of recurrent or persistent disease.^3,4^ The current tumor classifications do not allow for the identification of those patients that bear RAI refractory tumors and are, hence, hardly predictive for therapeutic success. Therefore, identification of prognostic markers with high predictive value is warranted in order to reliably estimate RAI treatment response and to optimize the therapeutic strategy for the individual patient. Furthermore, identification of these markers and understanding of the mechanisms involved in the dedifferentiation process could potentially facilitate the development of novel therapeutic approaches to improve the clinical response to RAI therapy.

One of the biological processes importantly involved in cancer development and progression is autophagy. Autophagy is a cellular machinery directing cellular metabolism and cell fate. Autophagy is activated in cells in case of nutrient deprivation, hypoxia and DNA damage to facilitate degradation of cytoplasmic components (e.g., damaged organelles and misfolded proteins), which are engulfed into autophagosomes and degraded following fusion of the autophagosomes with lysosomes to form autolysosomes.^5^ Recycling of degraded cellular components from autolysosomes provides a source of amino acids, nucleotides and lipids for adenosine triphosphate production and macromolecular synthesis. Autophagy has emerged as a key player in carcinogenesis and anticancer therapy resistance, including NMTC.^6,7^ In many tumor types, autophagy exhibits both promoting and inhibitory effects on tumor cell survival; on the one hand, autophagy inhibits tumor growth by inducing tumor cell cycle arrest, whereas, on the other hand, it is activated as a potent cell survival pathway promoting resistance to anticancer therapies.^8-10^

We and others have demonstrated in genetic and fundamental studies that autophagy itself and pathways that directly influence autophagy are involved in NMTC and its treatment response to RAI therapy.^11-13^ Of interest, also in other cancer types a clear association between autophagy activity, tumor aggressiveness and patient outcome has been demonstrated.^14,15^ However, no studies have been performed on the relationship between markers of autophagy with malignant transformation of thyroid follicular cells and sensitivity to RAI therapy in a large cohort of NMTC patients.

Punctate and diffuse cytoplasmic staining of LC3 (microtubule-associated protein 1 light chain 3; Atg8 in yeast) is well established as a marker for autophagy. LC3 is a constitutively expressed protein involved in autophagosome assembly.^16-18^ Depending on the degree of autophagy activity, LC3 proteins have different subcellular localizations. In the case of low autophagy activity, most of LC3 is diffusely present in the cytoplasm, designated as LC3-I. During active autophagy, however, cytoplasmic LC3-I is conjugated to phosphatidylethanolamine and incorporated into the lipid membrane of phagophores, the precursor to autophagosomes, as LC3-II. Consequently, LC3-II is localized to the cytosolic and luminal surfaces of mature, double-membraned autophagic vesicles that microscopically appear as intracellular puncta. At a later stage of the autophagic flux, i.e. fusion of the autophagosome with the lysosome, LC3-II on the autophagosome cytosolic surface is delipidated to LC3-I and recycled into the cytosol, whereas luminal LC3-II that was present on the luminal surface is degraded.^19^

In the present study, we hypothesized that the degree of LC3-I expression on the one hand and autophagy activity as reflected by the number of LC3-II-positive puncta on the other is different in patients with either benign or malignant thyroid lesions and that it correlates with the therapeutic efficacy of RAI treatment in NMTC patients. For this, expression of both unconjugated LC3-I and conjugated LC3-II has been assessed on tissue sections derived from benign thyroid tissues (Graves, goiter, follicular adenoma) and thyroid malignancies (papillary, follicular and anaplastic NMTC) and was compared to normal thyroid tissue. Moreover, the extent of autophagy expression and activity has been examined in dedifferentiated and RAI refractory non-medullary thyroid tumors vs. tumors that were well differentiated and exhibited RAI avidity.

## Results

### Validation of LC3 immunofluorescent staining with transmission electron microscopy and LC3 immunoblotting

**Figure 1.**
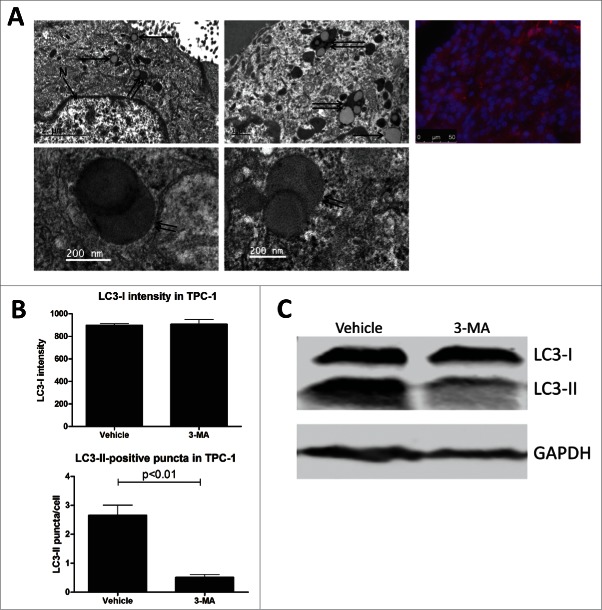
Validation of immunofluorescent LC3 staining and comparison of diffuse LC3-I intensity with LC3-II-positive puncta scores. (A) Comparison of autophagosome detection by transmission electron microscopy (left and middle panels) or by LC3 immunofluorescent staining (right panel). Arrows indicate autophagosomes alone, double arrows indicate colocalization of autophagosomes with lysosomes. N, nucleus. (B) LC3-I intensity and LC3-II-positive puncta scoring of TPC-1 cells treated with vehicle or 3-methyladenine (3-MA, 10 mM) for 4 h. Data have been generated by quantitative analysis with FIJI software. P-values have been calculated by the Mann-Whitney *U* test. (C) LC3 western blot of TPC-1 cells treated with dimethyl sulfoxide vehicle or 3-methyladenine for 4 h. GAPDH staining was performed to serve as loading control.

Transmission electron microscopy is regarded as the gold standard for visualizing autophagosomes. In order to validate immunofluorescent LC3-II staining for reliable quantification of autophagy activity, electron microscopy images were generated in parallel from fresh material obtained from 2 PTC patients. Representative pictures are depicted in Figure 1A, demonstrating concordant LC3-II-positive puncta scoring generated by both methods in the same tissue specimen. Of note, numerous double-membraned autophagosomes were detected undergoing lysosomal fusion, confirming active autophagy. Of note, most of the detected autophagosomes appeared to contain colloid. Additional validations were performed on the non-medullary thyroid cancer cell line TPC-1 that was treated with either vehicle or with 10 mM 3-methyladenine (3-MA, an autophagy inhibitor), which demonstrated corresponding changes in LC3-II-positive puncta and left LC3-I intensity unaffected (Fig. 1B). These results were confirmed by LC3 immunoblotting (Fig. 1C).

### Comparison of diffuse LC3-I intensity and number of LC3-II-positive puncta scores across different tissue subgroups and comparison with SQSTM1 expression

**Figure 2.**
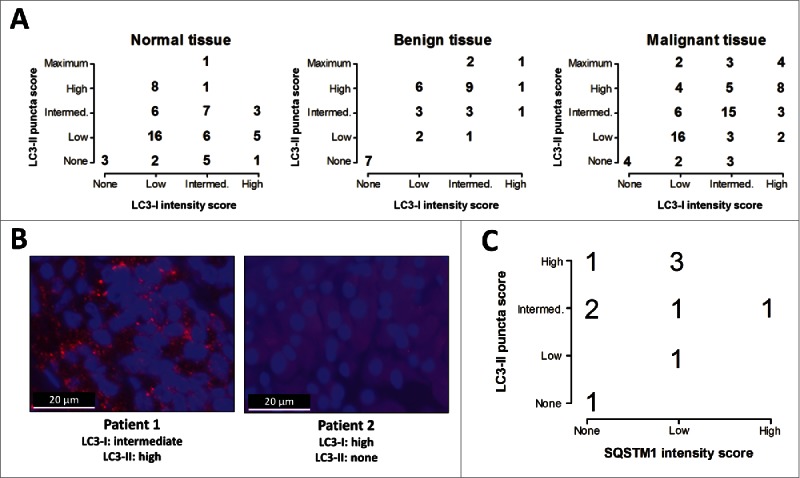
Distribution of LC3 scoring in normal, benign and malignant tissue groups and its correlation with SQSTM1 expression. (A) Cross-tabulations of diffuse LC3-I intensity and LC3-II-positive puncta scores within the normal, benign and malignant tissue groups. Intermed., Intermediate. (B) Examples of LC3 staining patterns and autophagy scoring in tissue samples of 2 non-medullary thyroid cancer patients (1000x magnification). (C) Correlation between number of LC3-II-positive puncta with SQSTM1 expression in non-medullary thyroid cancer tissue specimens (N = 10).

To compare the obtained semiquantitative scores for diffuse LC3-I staining intensity with the observed number of LC3-II-positive puncta within and across different tissue subgroups representing either normal, benign or malignant thyroid tissue, cross-tabulations were generated (Fig. 2A). These comparisons indicate that elevated expression of LC3-I was not necessarily coexistent with a high number of LC3-II-positive puncta, as exemplified in Fig. 2B; in the patient tissue depicted in the left panel, intermediate LC3-I intensity and a high number of LC3-II-positive puncta could be observed. In contrast, in the right panel, LC3-I intensity was high although LC3-II-positive puncta were nearly absent, scored as “0: none,” indicating minimal autophagy activity. LC3-I expression and incorporation of LC3-II into phagophores are therefore distinctly regulated processes, as reported previously.^20,21^ To assess whether the degree of LC3-II staining was related to SQSTM1 expression, 10 of the analyzed NMTC specimens were stained for both markers. No significant correlations were observed (Fig. 2C).

### Association of diffuse LC3-I intensity and LC3-II-positive puncta with thyroid pathology

**Figure 3.**
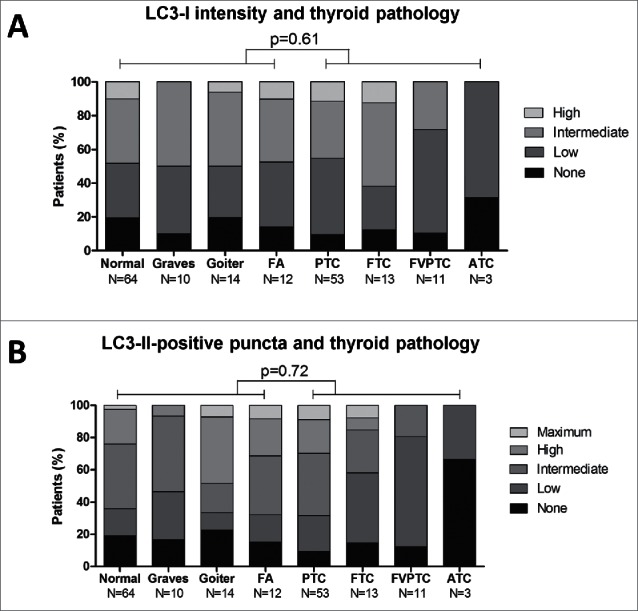
Distribution of diffuse LC3-I intensity (A) and LC3-II-positive puncta (B) scores divided by tissue group (normal and benign versus malignant) and subdivided by thyroid pathology. P-values were generated by χ^2^ tests. FA, follicular adenoma; PTC, papillary thyroid cancer; FTC, follicular thyroid cancer; FVPTC, follicular-variant papillary thyroid cancer; ATC, anaplastic thyroid cancer.

In order to examine the role of autophagy expression and activity in benign versus malignant thyroid pathology, the degree of diffuse LC3-I intensity and the number of LC3-II-positive puncta was assessed in both benign (normal thyroid tissue, Graves disease, goiter, follicular adenoma) and malignant lesions (PTC, FTC, FVPTC, ATC). After the assessment of autophagy scores, the distribution of these scores was analyzed in all subgroups of either benign or malignant thyroid lesions. The results indicate that neither autophagy expression (i.e., degree of diffuse LC3-I intensity) nor activity scores (i.e., number of LC3-II-positive puncta) were discriminative for benign vs. malignant tissues, meaning that this pathological dichotomy was not reproduced by the degree of either diffuse LC3-I intensity or LC3-II-positive puncta (Fig. 3). Moreover, the distribution of autophagy scores was similar in all analyzed subgroups, with the exception of the small group of ATC patients, and no statistically significant differences could be demonstrated between any combinations of 2 randomly chosen subgroups (data not shown).

### Association of diffuse LC3-I intensity and LC3-II-positive puncta with RAI uptake

**Figure 4.**
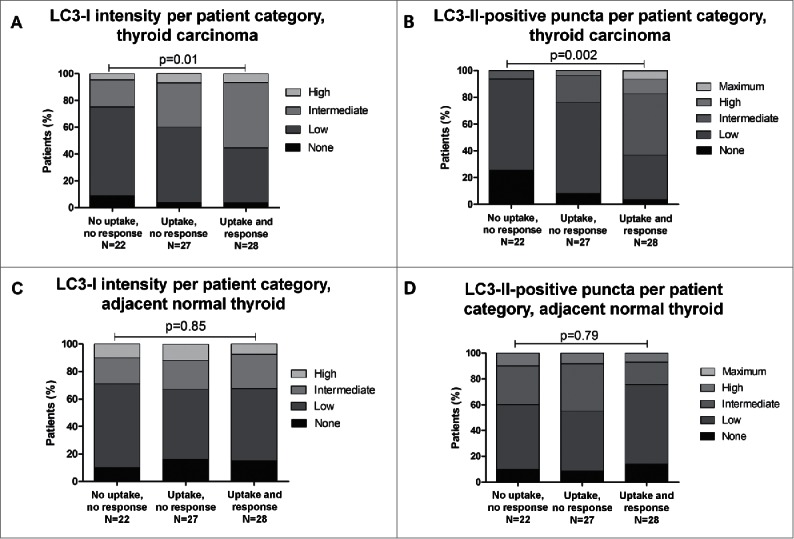
Distribution of diffuse LC3-I intensity and LC3-II positive puncta scores within the malignant thyroid tissue group (A, B; N = 77) and adjacent normal thyroid tissue (C, D; N = 77) and its correlation with uptake of and clinical response to radioactive iodine treatment in the corresponding non-medullary thyroid carcinoma patients. P-values were generated by χ^2^ tests.

Within the group of differentiated NMTC tissues comprising tumor and normal thyroid material obtained from PTC, FTC and FVPTC patients, data have been gathered on the extent of RAI uptake and the success rate of RAI ablation therapy in the corresponding tumors and surrounding normal thyroid tissue. Strikingly, both diffuse LC3-I intensity (P = 0.01, OR = 3.13 [95%CI 1.91–5.12]) and the number of LC3-II-positive puncta (P = 0.002, OR = 5.68 [95%CI 3.02–10.05]) were associated with clinical response to RAI (Fig. 4A, B). Specifically, tumors that had a poor response to RAI also displayed low LC3-I expression and a strongly reduced number of LC3-II-conjugated autophagosomes. Conversely, tumors characterized by high RAI uptake harbored significantly higher LC3-I expression and elevated numbers of LC3-II-positive puncta. No such associations were apparent in normal thyroid tissues surrounding NMTC tumors, suggesting a distinctive role for autophagy in maintaining expression of differentiation markers in thyroid malignancies as compared to physiological thyroid follicular cells (Fig. 4C, D).

### Association of iodide uptake, LC3-I expression and LC3-II positive puncta with membranous SLC5A5 expression

**Figure 5.**
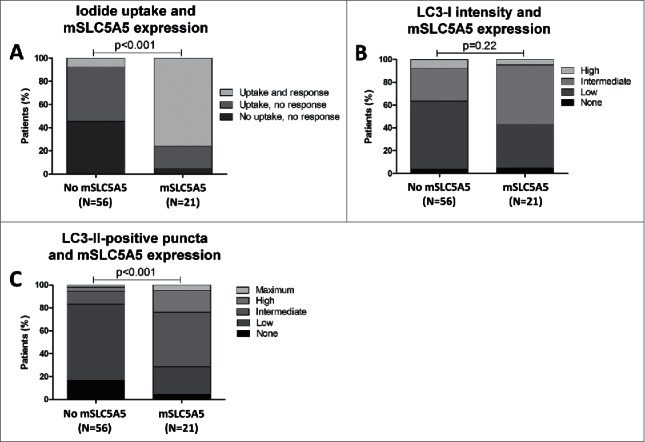
Correlation of membranous SLC5A5 (mSLC5A5) expression with uptake of and clinical response to radioactive iodine treatment (A), with diffuse LC3-I intensity (B) and with LC3-II-positive puncta scores (C) in non-medullary thyroid carcinoma tissues (N = 77). P-values were generated by χ^2^ tests.

Given the differential capacity in iodide uptake between tumors with either high or low autophagy activity, we hypothesize that a relationship could exist between SLC5A5 expression on the cell surface of thyroid tumor cells and autophagy. To examine this possibility, data on membranous SLC5A5 expression that were obtained for the same tissue specimens in a previous study^22^ and its potential association with LC3-I expression and number of LC3-II-positive puncta were analyzed. First, the degree of membranous SLC5A5 expression was correlated with the RAI uptake capacity and clinical response to RAI of the investigated tumors, which demonstrated that the presence of membranous SLC5A5 expression strongly predicted the clinical response to RAI therapy, as expected (P < 0.001, OR = 9.15 [95%CI 6.43–13.56]). Next, it was analyzed whether autophagy was related to membranous SLC5A5 expression. Indeed, membranous SLC5A5 expression was diminished in tumors with low autophagy activity whereas in tumors with membranous SLC5A5 expression, activity of the autophagy machinery, i.e., high numbers of LC3-II-positive puncta, was strongly elevated (P<0.001, OR = 7.71 [95%CI 4.15–11.75]). Interestingly, this association was not observed between membranous SLC5A5 expression and intensity scores of diffuse LC3-I expression (P = 0.22), suggesting a less important role for unconjugated LC3-I as compared to phagophore/autophagosome-associated LC3-II in maintaining a differentiated state of NMTC (Fig. 5).

### Association of autophagy scoring with clinical characteristics

To assess whether autophagy expression and activity are related to patient characteristics and outcome of the disease, autophagy scores were correlated with clinical parameters including tumor-node-metastasis (TNM) staging, cumulative RAI dose required to reach remission and disease persistence and recurrence rates. Whereas no significant associations were observed between clinical parameters and unconjugated LC3-I (Table 1), the number of LC3-II-positive puncta was clearly elevated in patients with low cumulative RAI dose, high remission rates and low recurrence rates during follow-up (Table 2).

**Table 1.** Correlation between patient characteristics and category of autophagy expression score in the corresponding NMTC tumor tissues (diffuse LC3-I intensity).

		Category autophagy expression (diffuse LC3-I intensity)				
Patient characteristics		None (N=3)	Low (N=28)	Intermediate (N=29)	High (N=17)	P-value*
Age at diagnosis in years (mean [±SD ])		46.9 (±15 .2)	52.2 (±15 .4)	50.2 (±17 .0)	51.9 (±17 .2)	0.832
Gender (Female/Male)		2/1	19/9	20/9	11/6	0.881
T-stage	T1	—	1 (3.6%)	2 (6.9%)	—	0.277
	T2	—	4 (14.3%)	3 (10.3%)	2 (11.8%)	
	T3	1 (33.3%)	5 (17.9%)	3 (10.3%)	4 (23.5%)	
	T4	2 (66.7%)	16 (57.1%)	18 (62.1%)	11 (64.7%)	
	Tx	—	2 (7.1%)	3 (10.3%)	—	
N-stage	N0	2 (66.7%)	17 (60.7%)	15 (51.7%)	6 (35.3%)	0.373
	N1	1 (33.3%)	7 (25.0%)	11 (37.9%)	8 (47.1%)	
	Nx	—	4 (14.3%)	3 (10.3%)	3 (17.6%)	
M-stage	M0	3 (100.0%)	17 (60.7%)	22 (75.9%)	10 (58.8%)	0.835
	M1	—	8 (28.6%)	5 (17.2%)	5 (29.4%)	
	Mx	—	3 (10.7%)	2 (6.9%)	2 (11.8%)	
Nr. RAI treatments	0-1	2 (66.7%)	15 (53.6%)	17 (58.6%)	10 (58.8%)	0.146
	≥2	1 (33.3%)	13 (46.4%)	12 (41.4%)	7 (41.2%)	
Cumulative RAI dose	<100 mCi	—	9 (32.1%)	10 (34.5%)	9 (52.9%)	0.089
	100-200 mCi	1 (33.3%)	4 (14.3%)	12 (41.4%)	4 (23.5%)	
	>200 mCi	2 (66.7%)	15 (53.6%)	7 (24.1%)	4 (23.5%)	
Disease after ablation	Remission	2 (66.7%)	15 (53.6%)	17 (58.6%)	10 (58.8%)	0.329
	Persistent	1 (33.3%)	13 (46.4%)	12 (41.4%)	7 (41.2%)	
Disease status during follow-up	Remission	2 (66.7%)	17 (60.7%)	12 (41.4%)	11 (64.7%)	0.517
	Persistent	1 (33.3%)	10 (35.7%)	16 (55.2%)	6 (35.3%)	
	Recurrent	—	1 (3.6%)	1 (3.4%)	—	

* P-values are generated by comparing group “none + low” with group “intermediate + high."

**Table 2.** Correlation between patient characteristics and category of autophagy activity score in the corresponding NMTC tumor tissues (LC3-II-positive puncta).

		Category autophagy activity (LC3-II positive puncta)					
Patient characteristics		None (N=7)	Low (N=20)	Intermediate (N=24)	High (N=17)	Maximum (N=9)	P-value*
Age at diagnosis in years (mean [± SD])		49.7 (±17 .7)	52.4 (±16 .6)	51.0 (±15 .6)	49.9 (±13 .6)	47.6 (±11 .4)	0.915
Gender (Female/Male)		4/3	14/6	17/7	11/6	6/3	0.845
T-stage	T1	—	2 (10.0%)	1 (4.2%)	—	—	0.405
	T2	1 (14.3%)	3 (15.0%)	4 (16.7%)	1 (5.9%)	—	
	T3	1 (14.3%)	1 (5.0%)	3 (12.5%)	3 (17.6%)	5 (55.6%)	
	T4	4 (57.1%)	12 (60.0%)	15 (62.5%)	12 (70.6%)	4 (44.4%)	
	Tx	1 (14.3%)	2 (10.0%)	1 (4.2%)	1 (5.9%)	—	
N-stage	N0	3 (42.9%)	8 (40.0%)	11 (45.8%)	12 (70.6%)	6 (66.7%)	0.124
	N1	4 (57.1%)	9 (45.0%)	7 (29.2%)	5 (29.4%)	2 (22.2%)	
	Nx		3 (15.0%)	6 (25.0%)	—	1 (11.1%)	
M-stage	M0	6 (85.7%)	16 (80.0%)	13 (54.2%)	10 (58.8%)	7 (77.8%)	0.111
	M1	1 (14.3%)	3 (15.0%)	8 (33.3%)	4 (23.5%)	2 (22.2%)	
	Mx	—	1 (5.0%)	3 (12.5%)	3 (17.6%)	—	
Nr. RAI treatments	0-1	2 (28.6%)	7 (35.0%)	13 (54.2%)	13 (76.5%)	9 (100.0%)	**0.014**
	≥2	5 (71.4%)	13 (65.0%)	11 (45.8%)	4 (23.5%)	—	
Cumulative RAI dose	<100 mCi	1 (14.3%)	5 (25.0%)	9 (37.5%)	8 (47.1%)	5 (55.6%)	**0.026**
	100-200 mCi	3 (42.9%)	3 (15.0%)	5 (20.8%)	7 (41.2%)	3 (33.3%)	
	>200 mCi	3 (42.9%)	12 (60.0%)	10 (41.7%)	2 (11.8%)	1 (11.1%)	
Disease after ablation	Remission	2 (28.6%)	7 (35.0%)	13 (54.2%)	14 (82.4%)	8 (88.9%)	**0.031**
	Persistent	5 (71.4%)	13 (65.0%)	11 (45.8%)	3 (17.6%)	1 (11.1%)	
Disease status during follow-up	Remission	1 (14.3%)	5 (25.0%)	14 (58.3%)	15 (88.2%)	7 (77.8%)	**0.042**
	Persistent	5 (71.4%)	14 (70.0%)	10 (41.7%)	2 (11.8%)	2 (22.2%)	
	Recurrent	1 (14.3%)	1 (5.0%)	—	—	—	

* P-values are generated by comparing group “none + low” with group “intermediate + high + maximum."

## Discussion

In the present study, we demonstrated the relation between active autophagy and clinical response to RAI therapy in NMTC patients. High autophagy activity, as reflected by high numbers of LC3-II-positive puncta in the cytoplasm of thyroid tumor cells, was correlated in a proportional fashion with uptake of and clinical response to RAI and with higher remission rates, less tumor recurrences and less exposure to RAI therapy, at least in part by its association with functional SLC5A5 expression on the basal membrane of thyroid tumor cells. Consequently, in case of high autophagy activity, the iodide uptake machinery was mostly intact, facilitating proficient ^131^I accumulation that allowed for complete eradication of malignant thyroid follicular cells in tumor remnants and metastases present after thyroidectomy.

RAI treatment is part of the standard of care for NMTC patients and is applied after thyroid surgery in order to eradicate thyroid (tumor) remnants and small metastases. The major therapeutic challenge in a subset of these patients is the development of resistance to RAI therapy in malignant cells, leaving these patients at high risk of tumor recurrence and outgrowth of metastases. Although the exact cellular mechanisms involved in conferring RAI therapy resistance remain elusive, it has been well established that a process of dedifferentiation accompanies resistance, i.e., there is loss of thyroid-specific gene expression.^3,23^

In other tissues and in different contexts, autophagy has been demonstrated to play a significant role in determining cell fate by directing pathways of proliferation and differentiation.^24,25^ Our previous studies have shown that inhibiting the MTOR kinase, a central player in the pathogenesis of NMTC, leads to restored SLC5A5 expression and iodide uptake in NMTC cell lines.^12^ One of the main downstream effects of MTOR is inhibition of autophagy, meaning that in the case of MTOR inhibition, autophagy becomes active and could therefore be responsible for the observed redifferentiation in these cell lines. Other direct and indirect indications that support an important role for autophagy are that highly active MTOR signaling, consequently low autophagy activity, and genetic variation in autophagy genes are associated with poor prognosis of NMTC patients and with a decreased ability for iodide uptake.^11,26-29^

By comparing LC3 scoring between tumor tissue and adjacent normal thyroid tissue, striking differences were observed. Whereas active autophagy predisposes to high uptake and to effective clinical responses to RAI therapy, this relationship was not observed in adjacent normal thyroid tissue surrounding these tumors. These differences might provide additional insights into the differential role of autophagy in malignant cells as opposed to normal thyroid follicular cells; cells that have been subjected to malignant transformation could deploy the autophagy pathway to maintain their differentiated state, whereas in normal thyroid cells the significance of autophagy to sustain thyroid-specific gene expression remains unapparent, most probably because the differentiation signature is not threatened by aberrant signaling evoked by oncogenic pathways.

In contrast to the profound association of autophagy activity with the clinical success rate of RAI therapy, no significant associations were observed in the comparison of LC3 scoring with either normal, benign or malignant thyroid tissues. These observations suggest that the absence or presence of active autophagy is not an independent driving force in malignant transformation of thyroid follicular cells, but might rather represent an important effect modifier of the clinical course of NMTC. In line with this, although only 3 ATC patients were analyzed, all displayed low autophagy expression and activity, confirming the inverse relationship between autophagy and NMTC aggressiveness. It remains to be determined whether the degree of autophagy activity also predisposes to a differential clinical course of benign thyroid disease, which is beyond the scope of the current study.

Identification of molecular players and biological mechanisms underlying the beneficial effects of autophagy on maintaining differentiation of NMTC cells requires further in-depth analysis of autophagy modulation in in vitro studies on NMTC cell lines and delineation of molecular signatures associated with differential expression and activity of autophagy in human tumor material. From the present study it can be deduced that these represent mechanisms most likely acting upon the regulation of autophagy activity rather than LC3 protein expression. As an alternative mechanism, increased numbers of LC3-II-positive puncta could also reflect impaired autophagosome maturation, which is indistinguishable from increased autophagic flux in fixated human tissue specimens. No clear association of LC3-II with SQSTM1 expression was observed, indicating that SQSTM1 could predominantly be regulated by other mechanisms, as previously reported.^30,31^ Another interesting observation is that most of the autophagosomes detected by electron microscopy appeared to contain colloid, a natural component of thyroid follicles located at the apical side of follicular cells. Future studies are warranted to confirm this and to explore its significance in thyroid carcinogenesis.

In recent years, several anticancer therapies have been tested for their ability to increase the therapeutic success of RAI therapy by inducing redifferentiation in NMTC. Of these, the selective BRAF^V600E^ inhibitor dabrafenib and the MAP2K/MEK inhibitor selumetinib display beneficial effects in small cohorts of RAI-refractory NMTC patients, of which 60% exhibit partial responses or stable disease after treatment with either dabrafenib or selumetinib followed by ^131^I RAI ablation therapy.^32,33^ It is well established that cellular signaling through oncogenic kinases, including BRAF and MAP2K, intertwine with autophagy pathways.^34-37^ Based on these considerations and the important role of autophagy in promoting a well-differentiated state in NMTC cells as suggested by the present study, one could envision that inhibitors of BRAF and MAP2K kinases lead to concomitant modulation of autophagy activity, which could thereby represent a critical factor influencing their beneficial clinical effects. Importantly, subsequent analyses are warranted in order to assess whether the magnitude of the observed clinical response to BRAF and MAP2K inhibitors in the respective studies is correlated with the degree of autophagy activity in responding and nonresponding thyroid tumors.

In conclusion, the present findings have important implications for understanding RAI therapy resistance in NMTC. They provide evidence for active autophagy as a predictive marker for maintained cellular quality control and counteracted tumor progression and aggressiveness, as also demonstrated in other tumor types and cancer models.^38-40^ Furthermore, it indicates that decreased autophagy activity is accompanied by loss of thyroid-specific gene expression and by diminished expression of SLC5A5 on the basal membrane of malignant thyroid follicular cells.

## Materials and methods

### Patients

Retrospectively, 180 histological samples from surgically removed thyroid lesions representing 7 different histological thyroid disorders and adjacent normal thyroid tissue were obtained from the pathological archive of the Leiden University Medical Center, The Netherlands. Ethical approval was obtained for all analyzed patient tissue specimens. We randomly selected 100 benign thyroid tissue samples (normal, N = 64; Graves disease, N = 10; multinodular goiter, N = 14; and follicular adenoma, all microfollicular, N = 12). In total 80 NMTC tissue samples (papillary TC [PTC], N = 53; follicular TC [FTC], N = 13; follicular variant of PTC [FVPTC], N = 11; anaplastic TC [ATC], N = 3) were selected based on their clinical response to RAI therapy in order to include sufficient numbers of both responding and nonresponding patients. All original histological diagnoses were reviewed by 2 independent observers. Patient data on demographic and clinical characteristics including tumor histology and TNM stage at diagnosis and information on tumor treatment and follow-up, i.e. the number of RAI therapy sessions and cumulative RAI dose were retrieved from the patient's medical records. Primary treatment of NMTC patients consisted of total or near-total thyroidectomy in all of the patients and modified radical neck dissections in patients with confirmed nodal metastases. This was followed by ablation with RAI (^131^I) of residual thyroid tissue after surgery. If necessary, patients were treated multiple times with RAI to reach remission. Initial cure was defined as undetectable thyroid-stimulating hormone-stimulated thyroglobulin (TG) in the absence of anti-TG antibodies or no evidence of loco-regional disease or distant metastasis on post therapeutic whole-body iodine scintigraphies and/or neck ultrasonographic examinations at 6–12 mo after RAI ablation. Disease status during follow-up was defined as remission in case of undetectable TG in the absence of anti-TG antibodies and no evidence of loco-regional disease or distant metastases at the last follow-up visit. Tumor recurrence was defined as new evidence of loco-regional disease or distant metastasis, also comprising biochemical recurrence based on TG positivity, more than 6 mo after successful primary therapy. Persistent disease was defined as detectable TG and/or evidence of loco-regional disease or distant metastases.

### Transmission electron microscopy

Fresh NMTC tissue was cut into small blocks and fixed in 2% buffered glutaraldehyde for at least 24 h. The tissue blocks were washed with 0.1M cacodylate buffer and post-fixed in 2% buffered osmium tetroxide for 1 h. The samples were dehydrated in a sequence of ethanol and propylene oxide, followed by EMbed 812 (Electron Microscopy Sciences, 14120) with propylene until it was pure EMbed 812 resin. The tissue samples were embedded in EMbed 812. Ultrathin sections were cut on a Leica Ultracut and stained with uranyl acetate and lead citrate. Photographs were taken on a Jeol 1400 electron microscope at 60 kV.

### Tissue microarrays

Formalin-fixed, paraffin-embedded blocks routinely prepared from the surgical specimens of thyroid tumors were selected for this study. Representative areas containing tumor or adjacent normal tissues were identified by a pathologist (HM). Triplicate tissue cores with a diameter of 0.6 mm were taken from each specimen (Beecher Instruments, Silver Springs, MD, USA) and arrayed on a recipient paraffin block, using standard procedures.^22,41^

### LC3 immunofluorescence and SQSTM1 immunohistochemistry

Four-micrometer consecutive tissue sections were cut from each arrayed paraffin block and prepared on pathological slides. The sections were deparaffinized in xylene and rehydrated in 100, 96 and 70% ethanol, successively. Subsequently, nonspecific binding sites were blocked by incubation with 20% normal goat or mouse serum (DAKO, X0907 or X0910, respectively) in phosphate-buffered saline (PBS; Fresenius Kabi GmbH, M090001). Then the sections were incubated with the polyclonal rabbit-anti-human LC3B primary antibody (1:200; Abcam, ab48394) or SQSTM1 primary antibody (1:400; BD Biosciences, 610832) diluted in PBS + 1% BSA (Sigma, A9418) overnight at 4°C. After 3 washing steps with PBS, LC3-stained sections were incubated for 1 h with goat-anti-rabbit Alexa Fluor 647 secondary antibody (ThermoFisher Scientific, A-21245) diluted 1:100 in PBS + 1% BSA. Sections were mounted with Vectashield containing diamidino-2-phenylindole (Vector Laboratories, H-1200). Negative controls were stained with the primary antibody omitted. Stainings were observed and scanned by a confocal laser-scanning microscope with 1000x magnification (Zeiss LSM700, Oberkochen, Germany). SQSTM1 stained sections were incubated with horseradish peroxidase conjugated secondary antibody (DAKO, P0161) 1:500 diluted in PBS + 1% BSA. The endogenous peroxidase activity was blocked with 3% H_2_O_2_ in methanol for 15 min at room temperature. Furthermore, because tumor-like tissues contain endogenous biotin, this was blocked in the tissue sections by an avidin-biotin blocking kit according to the manufacturer's protocol (Vector Laboratories, SP-2001). The ABC–horseradish peroxidase complex (Vector Laboratories, PK-6101), 1:200 diluted in PBS, was applied to the sections for 30 min at room temperature. The substrate solution was added for 5 min at room temperature: 0.5 ml of diaminobenzidene in 9.5 ml of PBS and 10 μl of H_2_O_2_. Tissues were counterstained with hematoxylin for 30 sec at room temperature. Slides were dehydrated with consecutive incubation in 70, 96 and 100% of alcohol and xylene (2 times) for 5 min each step. Sections were mounted in Permount (ThermoFisher Scientific, SP15–500).

### LC3-I intensity and LC3-II-positive puncta scoring

A semiquantitative assessment of immunohistochemical scoring was performed taking into account both the intensity of diffuse LC3-I staining (autophagy expression) and the number of fluorescent LC3-II-positive puncta (autophagy activity). The diffuse LC3-I staining intensity within cells was scored as follows: none, ‘0’; 0–30% (low), ‘1’; 30–70% (intermediate), ‘2’; and 70–100% (high), ‘3’. The number of fluorescent LC3-II-positive puncta was scored as none, ‘0’; low, ‘1’; intermediate, ‘2’; high, ‘3’; and maximum, ‘4’. Observers were blinded for clinical information during assessment of LC3-I and LC3-II staining. Scoring results were generated in triplicate for all patient tissues. Both parameters were assessed independently by 2 observers. In case of scoring discrepancies between triplicates or between the observers, which occurred in 18% of all specimens, tissue staining was re-evaluated and scoring consensus was reached. Only after all scoring data were collected, correlations with clinical data were performed.

### TPC-1 cell culture and LC3 analysis

The NMTC cell line TPC-1 (papillary, RET/PTC rearrangement) was obtained from the source previously described and was authenticated by short tandem repeat profiling.^42^ TPC-1 was cultured in DMEM medium (Invitrogen, 10566–016) supplemented with 10 µg/ml gentamicin, 10 mM L-glutamine, 10 mM pyruvate, and 10% fetal calf serum (ThermoFisher Scientific, 10270). Cells were grown on cover slips or in 6-well plates (5×10^6^ cells/well) and were incubated with dimethyl sulfoxide vehicle (Millipore, 317275) or with 3-methyladenine (10 mM, Sigma, M9281) for 4 h. For immunofluorescent LC3 staining, cells were fixed with 4% paraformaldehyde and permeabilized by cold methanol. For the remaining, fluorescent protein staining was performed according to the same protocol as described above. Quantitative scoring of LC3-I and LC3-II was performed with FIJI software. For immunoblotting of LC3 and GAPDH, cell lysates were subjected to protein electrophoresis on a 15% polyacrylamide gel. Protein transfer was performed on nitroglycerine membranes using the wet-blotting method (Bio-Rad, Hercules, CA, USA) and was followed by blocking, incubation with first (1:500; Abcam, ab48394) and second (ThermoFisher Scientific, A-21245) fluorescent antibody, each time using 5% (w/v) milk powder in Tris-buffered saline (Agilent Technologies, S3001) containing Tween 20 (Millipore, 655204). Fluorescent signal was detected by Odyssey equipment (Westburg, Leusden, The Netherlands).

### Statistical analysis

Associations of diffuse LC3-I intensity and number of LC3-II-positive puncta with thyroid pathology, clinical response to RAI therapy and membranous SLC5A5 expression (data obtained from previous study, see reference 22) were tested by 2-sided Chi-square tests. Furthermore, the following parameters were analyzed and correlated with autophagy scoring with the same statistical methods: (I) the tumor size at time of diagnosis was classified according to the sixth edition of the Union for International Cancer Control TNM classification;^43^ (II) the number of RAI treatments (including RAI ablation) as 0–1 treatments (e.g., no RAI ablation or exclusively ablation of thyroid rests after near-total thyroidectomy) or ≥2 treatments; (III) the cumulative RAI dosage as <100 mCi (<3.7 GBq), 101–200 mCi (3.8 – 7.4 GBq) or >200 mCi (>7.4 GBq) and (IV) the disease status after ablation and during follow-up expressed as remission, persistent or recurrent. Logistic regression was performed to generate odds ratios and 95% confidence intervals. For calculation of these values, comparison of LC3-I intensity scores was performed based on the formation of 2 scoring groups: (1) score none-low and (2) score intermediate-high, whereas for LC3-II-positive puncta scores this was generated by comparing scoring groups (1) score none-low and (2) score intermediate-high-maximum. Quantitative LC3 scoring of TPC-1 was statistically analyzed by Mann-Whitney *U* tests. A p-value below 0.05 was considered as statistically significant.

## Abbreviations

3-MA: 3-methyladenine
ABC: avidin-biotin complex
ATC: anaplastic thyroid carcinoma
ATG: autophagy related
BSA: bovine serum albumin
FTC: follicular thyroid carcinoma
FVPTC: follicular variant papillary thyroid carcinoma
LC3: microtubule associated protein 1A/1B light chain 3
MTOR: mechanistic target of rapamycin (serine/threonine kinase)
NMTC: non-medullary thyroid cancer
OR: odds ratio
PBS: phosphate-buffered saline
PTC: papillary thyroid carcinoma
RAI: radioactive iodine
RET: rearranged during transfection
SLC5A5/hNIS: solute carrier family 5 member 5
SQSTM1: sequestosome 1
TG: thyroglobulin
TNM: tumor-node-metastasis
